# Skin-Grafting Superseded by Skin-Propagation

**Published:** 1899-12

**Authors:** 


### SKIN-GRAFTING SUPERSEDED BY SKIN-PROPAGATION.

The ability of hæmatherapy to propagate new skin on the largest raw surface from a few minute and scattered points of graft skin, such as the patient or any one else can furnish from his own cuticle without inconvenience, while at the same time putting a stop to the pain, waste, and fever that are so terribly fatal in extensive lacerations or burns, is a discovery full of such blessed respite from agony and death, for the future, that we need not apologize for once more repeating the memorable first case.

The pain attendant upon skin grafting, both to the giver and the receiver, the difficulty of procuring the living human skin in sufficient quantity, and the tedious long period of suffering while the grafts are setting or failing to set—these render a revolution in this great life-saving process, that does away with all trouble, an event that one would expect to be hailed and celebrated the world over. Why not? Can any one tell?

